# Influence of Chitosan Ascorbate Chirality on the Gelation Kinetics and Properties of Silicon-Chitosan-Containing Glycerohydrogels

**DOI:** 10.3390/polym10030259

**Published:** 2018-03-02

**Authors:** Natalia O. Gegel, Yulia Yu. Zhuravleva, Anna B. Shipovskaya, Olga N. Malinkina, Irina V. Zudina

**Affiliations:** 1 Institute of Biochemistry and Physiology of Plants and Microorganisms, Russian Academy of Sciences, Entuziastov 49, Saratov 410049, Russia; Zhuravleva11.03@mail.ru; 2 Department of High Molecular Compounds, Saratov State University, Astrakhanskaya 83, Saratov 410012, Russia; Shipovskayaab@yandex.ru (A.B.S.); olga-malinkina@yandex.ru (O.N.M.); ivzudina@mail.ru (I.V.Z.)

**Keywords:** chitosan, l- and d-ascorbic acid, polysalt, chirality, silicon-chitosan-containing glycerohydrogels, biomimetic sol–gel synthesis, antibacterial activity, fibroblasts

## Abstract

The influence of the chirality of chitosan ascorbate on the gelation kinetics and the properties of hybrid silicon-chitosan-containing glycerohydrogels were studied with a deep estimation of the stereospecificity of chitosan polysalts with l- and d-ascorbic acid diastereomers and their biological effects. It has been established that l- and d-diastereomerically enriched chitosan ascorbates are characterized by a positive Cotton effect and differ in the wavelength of the maximum of the dichroic band (250 and 240 nm), as well as in the values of its specific ellipticity (21.8 × 10^5^ and 39.2 × 10^5^ deg·mL·dm^−1^·g^−1^), the sign of specific optical rotation (+ and −), the type of dispersion curves (anomalous and smooth), as well as the condensed phase morphology (anisodiametric particles with optical anisotropy and confocal domains of spherical shape, respectively). In the biomimetic sol-gel synthesis of silicon-chitosan-containing glycerohydrogels using silicon tetraglycerolate as a precursor, it was found that chitosan d-ascorbate retarded gelation. Thin congruent plates obtained from the corresponding glycerohydrogels based on chitosan d-ascorbate have higher mechanical strength and elasticity under uniaxial stretching and lower values of Young’s modulus. It has been shown that the systems based on chitosan d-ascorbate show the greatest antibacterial activity against *Staphylococcus aureus* 209P and *Escherichia coli* 113-13 and significantly promote the viability of normal human dermal fibroblasts. The results of our assessment of the biological properties of chitosan polysalts are unexpected, since ascorbic acid exhibits biological activity as its l-isomer only.

## 1. Introduction

The design of environmentally friendly, biocompatible, and biodegradable hydrogel systems is one of the topical scientific problems in the field of polymer-containing biomaterials [1,2]. Natural polysaccharides, in particular, the aminopolysaccharide chitosan, are considered as promising polymers for obtaining such forms [3,4]. Water-soluble derivatives of this polymer, in particular, the salts of chitosan with biologically-active organic acids (ascorbic, amino benzoic, aspartic acid, etc.) were most widely used in the development of hydrogel preparations for medical and biological purposes [5,6].

Chitosan belongs to the class of optically active (chiral) polymers. The presence of asymmetrically substituted carbon atoms (chiral centers) in its macromolecules leads to the appearance of the optical activity of one unit and, as a consequence, that of the macromolecule as a whole [7,8]. The ionic complexation of auxochromic amino groups with an organic acid, which occurs when water-soluble chitosan derivatives are formed, is accompanied by the formation of optically-active chromophores and affects the macrochain conformation, the morphology, and physicochemical properties of chitosan salts, thus expanding the spectrum of their biologically-useful properties [6,9]. Control of the chiral organization of the polymer opens up wide possibilities for the formation of materials not only with a pregiven stereostructure and properties on its basis, but also with controlled sites of complementary-specific interactions. For example, a correlation was established between the magnitude and sign of the specific optical rotation of chitosan acetate with its antibacterial action against *S. aureus* and *E. coli* [10,11]. Wen et al. [12] found the effect of chitosan on the enantioselective bioavailability of dichloroprop™ (a chiral herbicide) when *Chlorella pyrenoidosa* was cultured in its medium: in the presence of chitosan, the herbicidally-active (*R*)-enantiomer showed less toxicity than the ballast (*S*)-enantiomer did.

The biomimetic sol–gel technology is one of the relatively new and promising fields for designing biocompatible hybrid hydrogel materials [13,14,15]. The literature contains a sufficient number of publications devoted to the sol–gel synthesis of inorganic-organic polyfunctional hybrid structures based on chitosan and biogenic elements. The major body of these publications is devoted to obtaining and studying the structure and properties of biomimetic calcium-containing polysaccharide materials [13,16,17,18]. Silicon atoms are introduced into the structure of the inorganic precursor to improve the stability of the material in the chemically-active medium of the human body, to increase the bioactivity while preserving the biocompatibility inherent in the calcium polysaccharide-containing biomimetics [19]. Despite the fact that the biogenic micronutrient silicon is contained in the human body in a small amount (~10^−3^%), it is present in virtually all its organs and tissues. The connective, epithelial, bone, and skin tissues are the most rich in silicon [20].There are data on the sol–gel preparations of hybrid silicon-polysaccharide hydrogels using sodium silicate as a precursor, and chitin or chitosan as polysaccharides [14,21]. Other well-known precursors, namely, tetramethoxysilane and tetraethoxysilane, have also been used to prepare hybrid hydrogels [22,23]. Meanwhile, alcohols (methanol and ethanol) formed during their hydrolysis may cause polysaccharide precipitation, which significantly complicates their use in medical applications. Adverse byproducts (diols) are also formed in the synthesis of hybrid chitosan-silicon structures using glycidoxypropyltrimethoxysilane [24]. Shchipunov et al. [15] first proposed a water-soluble biocompatible precursor (tetra*kis*(2-hydroxyethyl)orthosilicate) for synthesizing silicon-chitosan-containing hydrogels, which makes it possible to carry out the sol–gel process under mild conditions. In this case, ethylene glycol released in hydrolysis and condensation reactions does not adversely affect chitosan. The use of such precursors as silicon glycerolates: tetra*kis* (2,3-dihydroxypropoxy) silane, and its combination with dimethyl*bis* (2,3-dihydroxypropoxy) silane in a 1.0:0.5 molar ratio for the non-catalytic sol–gel synthesis of chitosan-based hydrogels is also known [25].

We considered the biomimetic sol-gel synthesis of silicon-chitosan-containing glycerohydrogels using glycerol solutions of silicon tetraglycerolate and aqueous solutions of chitosan salts with organic (СН_3_СООН, HOOCCH_2_OH, and С_6_Н_8_О_6_) and inorganic (HCl) acids [19,26,27]. The influence of the concentration of the precursor and chitosan salt, the pH of the medium and the temperature of the sol–gel process on the gelation time of the multicomponent chitosan-containing system was estimated. It has been established that chitosan accelerates gelation in weakly-acidic media; in more acidic media, the kinetics of the process changes along a curve with a maximum, which may be due to various mechanisms of the reaction of silanol condensation before and after the isoelectric point. Electron microscopy has shown that biomimetic mineralization of the polysaccharide occurs in the formation of these hybrid hydrogels, and the hydrogel skeleton is a 3D network consisting of agglomerated supramolecular chitosan structures coated with a “shell” of amorphous SiO_2_ [26,27]. It is shown that, in practical terms, the salts of chitosan with pharmacopeic organic acids, in particular, with ascorbic ones, are the most promising to synthesize pharmacologically-active hydrogels. Despite the fact that ascorbic acid is characterized by the existence of several stereomeric forms (e.g., l- and d-ones), only the l-isomer (vitamin C) is most often used in studies. Meanwhile, the different reactivity of l- and d-ascorbic acid with chitosan, the differences in the parameters of the monoclinic cell of anhydrous crystals of chitosan l- and d-ascorbates described in [28], as well as the effects of stereocomplementarity of their spatial organization, may have a significant impact on both the general chirality of the polymeric system and the properties of the materials obtained on its basis, and the character of their biological effects.

The purpose of this work was to study the effect of chirality of chitosan ascorbate on the gelation kinetics and properties of hybrid silicon-chitosan-containing glycerohydrogels with a deep evaluation of the stereospecificity of chitosan polysalts with l- and d-ascorbic acid isomers and their biological effects.

## 2. Materials and Methods

### 2.1. Materials

The following reagents were used: chitosan hydrochloride (CS·HCl) with a viscosity average molecular weight ${\bar{M}}_{\eta }$ = 38 kDa, a degree of deacetylation DD = 80 mol %, and chitosan (CS) with ${\bar{M}}_{\eta }$ = 200 kDa, DD = 82 mol % (Bioprogress Ltd., Shchelkovo, Russia); poly(vinyl alcohol) (PVA) with a weight average molecular weight of 89–98 kDa and a 99% basic substance content (Sigma Aldrich, St. Louis, MO, USA); l-ascorbic acid (l-AscA) with a 99% basic substance (Meligen Corp., St. Petersburg, Russia), d-ascorbic acid (d-AscA) with a 98% basic substance (Khimreaktiv Corp., Moscow, Russia); l-menthol with a 99% basic substance (Alfa Aesar, Heysham, Lancs, UK); 95% ethyl alcohol (RFK Corp., Orel, Russia); tetraethoxysilane Si(OEt)_4_ (Ekos-1 Ltd., Moscow, Russia); glycerol (GlyOH, Vekton Ltd., St. Peterburg, Russia); distilled water and Milli-Q water. All reagents were chemical grade and used without further purification.

### 2.2. Methods of Preparation

#### 2.2.1. Solution Preparation

Aqueous solutions of l-AscA, d-AscA, and CS·HCl, as well as those of CS·HCl and CS in l-AscA and d-AscA, were prepared in a –NH_2_:AscA equimolar ratio and used in our experiments. All solutions with AscA were prepared in the absence of natural light. Experiments were carried out under identical conditions with freshly-prepared solutions of the substances used.

Solutions of l-AscA and d-AscA with concentrations within 0.1–7.0 g·(100 mL)^−1^ were prepared by dissolving the acid powder in the calculated amount of water at 20 ± 2 °C during 1 h. Aqueous solutions of CS·HCl with concentrations within 0.5–5.0 g·(100 mL)^−1^ were prepared by dissolving the polymer powder in the calculated amount of water at 20 ± 2 °C during 1 day. To prepare CS·HCl and CS solutions in l-AscA and d-AscA with concentrations within 0.1–7.0 g·(100 mL)^−1^, a sample of polymer powder was suspended in the calculated amount of water on a magnetic stirrer, followed by addition of an air-dry AscA powder. The system was left at 20 ± 2 °C for one day until complete dissolution. The pH of these aqueous solutions with a concentration of 0.5 g·(100 mL)^−1^ was: рН_l-AscA_ = 3.0, рН_d-AscA_ = 2.9, рН_CS·HCl_ = 4.9, рН_CS·HCl+l-AscA_ = рН_CS·HCl+d-AscA_ = 3.0, and рН_CS+l-AscA_ = рН_CS+d-AscA_ = 4.8; those with concentrations of 3–4 g(100 mL)^−1^: рН_CS·HCl_ = 3.5, рН_CS·HCl+l-AscA_ = рН_CS·HCl+d-AscA_ = 3.1, and рН_CS+l-AscA_ = рН_CS+d-AscA_ = 4.2.

A solution of l-menthol with a concentration of 5.0 g·(100 mL)^−1^ was prepared by dissolving a sample of air-dry l-menthol in 95% ethyl alcohol for 24 h.

Aqueous PVA solutions of a concentration of 10.0 g·(100 mL)^−1^ were prepared by suspending a sample of polymer powder in the calculated amount of water on a magnetic stirrer for 5 min, followed by 850 W microwave treatment in a Mars-5 laboratory microwave system (CEM Corporation, Matthews, NC, USA) for a during of 30–50 s.

The concentrations of the l-menthol and PVA solutions used were determined in preliminary experiments.

pH was measured on a Mettler Toledo Five Easy FE20 pH meter (Stützerbach, Germany).

Gravimetric measurements were carried out on Ohaus Adventurer AR 1530 scales (the weighing accuracy ±0.002 g).

#### 2.2.2. Precipitation

To obtain a precipitate, the aqueous CS·HCl solution in l-AscA or d-AscA with the concentrations of 0.5 and 1.0 g·(100 mL)^−1^ was poured into a Petri dish, then the alcoholic solution of l-menthol with the concentration of 5.0 g·(100 mL)^−1^ was added in a volume ratio of 1:1, the system was thoroughly mixed and left in the open air at 20 ± 2 °C for 48 h until the liquid component completely evaporated. The morphology of the precipitate was examined in polarized light (see Methods of examination).

#### 2.2.3. Synthesis of Organically-Modified Silicon

Silicon tetraglycerolate was synthesized by tetraethoxysilane transesterification in a polyol excess with no catalyst [25]:

Si(OEt)_4_ + 7GlyOH →Si(OGly)_4_·3GlyOH + 4EtOH.

At the first stage, esterification between tetraethoxysilane and glycerol was carried out at 70–80 °C under constant stirring until the phase boundary disappeared. At the second stage, the resulting free ethanol was distilled out from the reaction mixture at 80 °C under atmospheric pressure. The completion of this stage was controlled by the temperature lowering down to 73 °C and by the volume of the distilled ethanol as an azeotropic mixture (~75% of the theoretical value). At the third stage, the ethanol was removed from the system at 140 °C under 15 mm Hg during 3 h. The purity of the inorganic phase precursor was verified by IR spectroscopy by the absence of the signals of >С=О and >С=С< within 1705–1685 cm^−1^, characteristic of the product of thermal oxidation of glycerol (acrolein).

#### 2.2.4. Sol–Gel Synthesis of Glycerohydrogels

Silicon-chitosan-containing glycerohydrogels were formed as bulk monoliths and in the form of thin-film plates.

To synthesize glycerohydrogels in the form of bulk monoliths, mixtures of the CS·HCl and CS solutions in l-AscA and d-AscA with concentrations 3.0–7.0 g·(100 mL)^−1^ and Si(OGly)_4_·3GlyOH in 1:1–13:1 weight ratios were used. The mixtures were thoroughly stirred during 1–2 min to homogeneity and kept for the sol–gel process to proceed at 20 ± 2 °C. The gel point was fixed by the time of fluidity loss of the system by “turning the flask”. Glycerohydrogel plates were prepared by mixing the CS solutions in l-AscA and d-AscA with concentrations 3.0–7.0 g·(100 mL)^−1^, the PVA solution with the concentration of 10.0 g·(100 mL)^−1^ and Si(OGly)_4_·3GlyОН in a 4.5:1:0.5 weight ratio. The mixtures were thoroughly stirred for 4–5 min until homogeneity, placed on a horizontal polypropylene substrate at a rate of 0.4 mL/cm^2^ and allowed for sol–gel process to proceed at 20 ± 2 °C.

To express the component composition of the glycerohydrogel system under study (bulk monoliths, thin-film plates), the polymer/precursor weight ratio (*С*_CS·HCl_/*С*_Si_, *С*_CS_/*С*_Si_) was used.

### 2.3. Methods of Examination

#### 2.3.1. Spectroscopic Methods

Circular dichroism (CD) spectra were recorded on a Chirascan™ spectrometer (Applied Photophysics Ltd., Beverly, MA, USA) with a UV detector in the wavelength range λ = 200–600 nm at 25 °C. Thermostated quartz cells “Hellma Analytics” (Müllheim, Germany) were used. A 150 W xenon lamp was the light source. CD spectra were recorded in a scanning mode with a step of 1 nm and a detection time of 0.5 s, followed by subtraction of the baseline (the spectrum of the solvent Н_2_О). The CD signals obtained were processed using Pro-Data software (Applied Photophysics Ltd., Beverly, MA, USA), the spectra were not smoothed. The CD spectra were represented as the difference in the absorption of the left-hand and right-hand circular polarized light and expressed in units of specific ellipticity [θ]. Since the CD spectra of strongly dissociating chitosan salts were analyzed, the use of reduced values (for example, molar ellipticity) was not fairly justified. This is due to the possible existence of regions where the recorded spectral effect may be a superposition of signals. In our case, the spectral bands of chitosan ascorbate and free ascorbate anions may overlap in the wavelength region below 300 nm. In parallel with CD measurements, the UV absorption spectra of the solutions of the substances studied were monitored.

Optical rotation dispersion (ORD) spectra were recorded on an automatic SPU-E spectropolarimeter (RF) in the range λ = 280–710 nm at 25 °C. Thermostated cells with quartz windows (RF) were used. A high-pressure mercury lamp DRSh-250 (RF) was the light source. The experimental conditions were standard, and the error in measuring rotation angles did not exceed ±0.002°. The values of specific optical rotation [α] (see above) were used to plot the ORD curves. The concentration of working solutions was determined in special experiments, proceeding from the condition of no concentration dependence of [α] to exclude possible intermolecular interactions between chains. Every ORD curve was plotted from the results of three replicate experiments.

Specific ellipticity [θ] (deg·mL·dm^−1^·g^−1^) and specific optical rotation [α] (deg·mL·dm^−1^·g^−1^) were calculated from Equations (1) and (2), respectively:

(1) $${\left[\theta \right]}_{\lambda ,\mathrm{nm}}^{25{}^{\circ}C}=\frac{\Delta \theta \cdot 100}{C\cdot l},$$

(2) $${\left[\alpha \right]}_{\lambda ,\mathrm{nm}}^{25{}^{\circ}C}=\frac{\left(\alpha -\alpha_{0}\right)\cdot 100}{C\cdot l},$$

where Δθ is the measured angle of light ellipticity, deg; α and α_0_—the measured angles of optical rotation of the solution and solvent (H_2_O), respectively, deg; *C*—the concentration of the solution, g·(100 mL)^−1^; *l*—the optical path length, 1 dm. To record CD and ORD spectra, aqueous solutions of a concentration of 0.5 g·(100 mL)^−1^ were prepared. In our CD experiments, the stock solutions were diluted with Milli-Q distilled water at a 1:250 dilution.

IR spectra were recorded on a Vertex 70 v vacuum FTIR spectrometer (Billerica, MA, USA) with a PIKE GladiATR thermovariation and a resolution of 4 cm^−1^, using an average of 36 scans in the range of 4000–400 cm^−1^ by ATR. Spectra were processed by OPUS software. Vibrational absorption bands were assigned to functional groups by using conventional correlation tables [29].

^1^H and ^1^H–^13^C NMR spectra were recorded on a Bruker Avance II 600 spectrophotometer (Billerica, MA, USA) equipped with an inverted BBI wideband sensor, suppressing the signal of water. ^1^H–^1^H NMR spectra were recorded on a VARIAN-400 spectrophotometer (Palo Alto, CA, USA). In all experiments, an average of 32 scans with a relaxation time of 1 s was used in a frequency range of 5.4–2.0 ppm. D_2_O was used for sample preparation of the analyzed systems. The temperature was 40 °C.

#### 2.3.2. Elemental Analysis

Elemental analysis was carried out on a Vario Micro Cube analyzer (Elementar, Langenselbold, Germany). C, H, and Cl were analyzed in a flow of O_2_, N was analyzed in a flow of CO_2_. The error was ±0.5 wt %. Potentiometric titration was carried out on an automatic Mettler Tolledo G20 titrator (Stützerbach, Germany). Working solutions were prepared using standard HCl and NaOH titers.

#### 2.3.3. Polarization Microscopy

Optical observations in polarized light were performed on a LaboPol-2 (RF) polarization microscope with the polarizer and analyzer crossed. A halogen lamp (12 V, 30 W) served as the light source. Photos were obtained by a DMC 300 (3 Mpx) USB camera (Hangzhou, China).

#### 2.3.4. Mechanical Properties

Elastoplastic properties were evaluated on an Instron 3342 tensile machine (Darmstadt, Germany) used a 0.5-kN load cell in a uniaxial tensile mode at a speed of 10 mm/min. Based on the data obtained, stress-strain curves were plotted, from which the rupture stress (MPa), elongation at break (%) and Young’s modulus (MPa) were calculated. Physicomechanical characteristics were calculated taking into account the initial length and thickness of the sample. Six replicate experiments were performed.

### 2.4. Evaluation of Antibacterial Activity

Antibacterial activity was evaluated by diffusion to agar using the model of one Gram-positive (*S. aureus* 209P) and one Gram-negative (*E. coli* 113-13) strain of bacteria. Solutions with a concentration of 0.5 g·(100 mL)^−1^ were used. An aqueous solution of chlorhexidine bigluconate (Yuzhfarm Ltd., Krasnodar, Russia) with a concentrations of 0.05 g·(100 mL)^−1^ applied in medicine as an aseptic was used as the standard. Six wells with a diameter of 7 mm were made in a Petri dish with frozen meat-peptone agar containing a cell suspension of a 24-h bacterial test culture with a concentration of 10^8^ cells·mL^−1^. 0.1 mL of test solutions was added into three wells, and 0.1 mL of the standard solution was added into the three other wells. Zones of bacterial growth retardation were measured after 18–20 h of cultivation. The experiment was repeated three times. Antibacterial activity (*Ai*, %) was expressed in units of the reference concentration of the standard by the formula *Ai* = (*d_i_*/*d*_0_)·100%, where *d_i_* and *d*_0_ are the diameter of the growth inhibition zone of the test culture with the test solutions and the standard one, respectively.

### 2.5. Viability Test

#### 2.5.1. Cell Preparation

The hospital’s committee of ethics approved this study, and informed consent was obtained from all subjects. Primary normal human dermal fibroblasts (NHDF) were isolated from skin biopsy of a healthy young male donor (Municipal Clinical Hospital No. 7, Saratov, Russian)by sequential trypsin and collagenase digestion and were expanded in Dulbecco’s Modified Eagle Medium (DMEM, Sigma-Aldrich, St. Louis, MO, USA), containing 10% fetal bovine serum (FBS, Hyclone), 2 mM l-glutamine (Sigma-Aldrich, St. Louis, MO, USA), and 1% penicillin–streptomycin antibiotic antifungal cocktail (Sigma-Aldrich, St. Louis, MO, USA). Cell stocks were frozen in complete DMEM containing 10% dimethylsulfoxide and kept in liquid nitrogen until use. Cells were revived by thawing at 37 °C and were further propagated in DMEM complete. NHDF were used in passages 2–6. The media were replaced every three days, and the cells were maintained in a humidified incubator at 37 °C with 5% CO_2_. Cell cultures with 75–85% confluence were harvested using 0.25% trypsin (Life Technologies, Delhi, India) and counted with a hemocytometer.

#### 2.5.2. Cell Viability

NHDF cells were seeded into 96-well cell-culture plates at a cell density of 10^4^/well. After 24 h of cultivation, 200 μL of a solution of an initial concentration of 0.1 g (100 mL)^−1^ with dilutions of 1:10 and 1:14 (10^−2^ and 7 × 10^−3^ g·(100 mL)^−1^)were added into the plate and incubated overnight at 37 °C under 5% CO_2_. 100 μL of fresh medium and 10 μL of fluorescence dye Alamar Blue (Sigma-Aldrich, St. Louis, MO, USA) were added to each well. Intensity was measured by spectrophotometry (Gemini XPS Microplate Reader, Molecular Devices, Ramsey, MN, USA) by absorbance measurements at 560 nm and 635 nm after 4 h of incubation. The experiment showed the ability of metabolically-active cells to convert the Alamar Blue reagent into a fluorescent and colorimetric indicator [30].Ten parallel experiments were carried out.

### 2.6. Statistical Analysis

The results are presented as mean values ± standard deviation (*n* ≥ 3). The significant difference was evaluated by unpaired two-sample Student’s *t*-test. The difference was considered statistically significant when *p* < 0.05.

## 3. Results and Discussion

### 3.1. A Study of the Chirality of Diastereomerically-Enriched Chitosan Salts

When CS·HCl (CS) was dissolved in aqueous solutions of l- and d-AscA, diastereomerically-enriched chitosan complexes (hereinafter referred to as salts enriched with either l-AscA or d-AscA) of two types were formed, namely: the binary salts chitosan hydrochloride-l-ascorbate (CS·HCl·l-AscA) and chitosan hydrochloride-d-ascorbate (CS·HCl·d-AscA), and the monosalts chitosan l-ascorbate (CS·l*-*AscA) and chitosan d-ascorbate (CS·d-AscA). Polysalt formation was proven by FT-IR and NMR spectroscopy (by the presence of the absorption maximum at ~1600 cm^−1^ in the IR spectra and the presence of the signal within 3.15–3.18 ppm in the NMR spectra, which prove the formation of protonated amino groups), elemental analysis (the gross formula and the reduced molecular weight of the monomer unit calculated with allowance for the deacetylation degree of the sample were: С_6.8_H_13.6_NCl_0.7_O_4.8_·Н_2_О and 228.9 Da for CS·HCl·AscA, С_10.4_H_17.1_NO_8.5_·Н_2_О and 309.9 Da for CS·AscA, respectively), and potentiometric titration (by the amount of acid bound to the amino groups of chitosan molecules).

**Figure 1:**
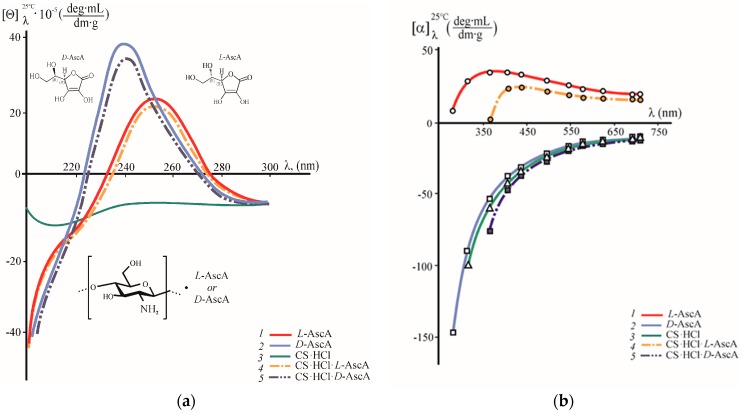

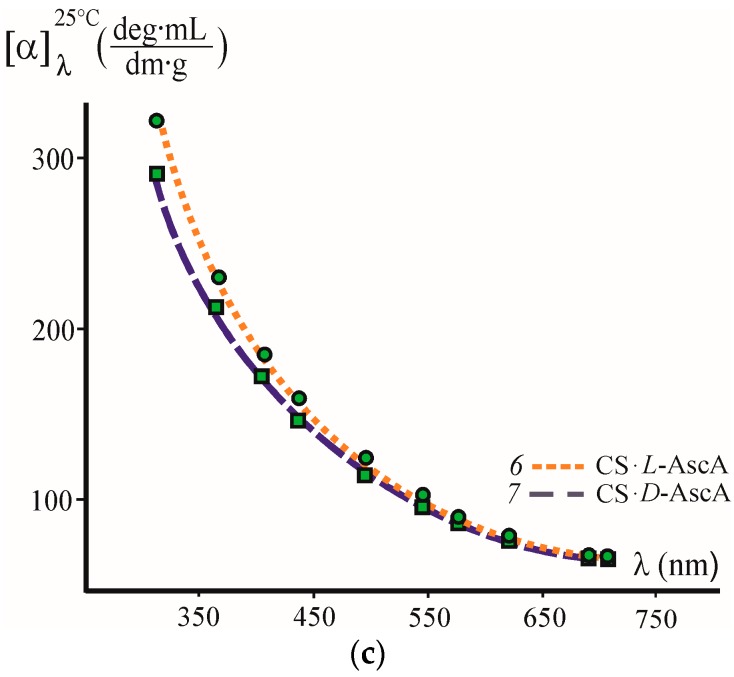
CD spectra (**a**) and ORD curves (**b**,**c**) of aqueous solutions of l-AscA (*1*), d-AscA (*2*), CS·HCl (*3*), CS·HCl·l-AscA (*4*), CS·HCl·d-AscA (*5*), CS·l-AscA (*6*), and CS·d-AscA (*7*). The polysalts CS·HCl·AscA and CS·AscA were obtained at a 1:1 CS·HCl(CS):AscA molar ratio. The pH of the aqueous solutions of the concentration 0.5 g·(100 mL)^−1^ was: рН_l-AscA_ = 3.0, рН_d-AscA_ = 2.9, рН_CS·HCl_ = 4.9, рН_CS·HCl+l-AscA_ = рН_CS·HCl+d-AscA_ = 3.0, and рН_CS+l-AscA_ = рН_CS+d-AscA_ = 4.8.

CD and ORD spectroscopy was used to study the chirooptical properties of the diastereomerically-enriched chitosan polysalts with l- and d-ascorbate anions. Figure 1a shows the CD spectra of aqueous solutions of the chitosan binary salts CS·HCl·l-AscA and CS·HCl·d-AscA. The CD spectra of aqueous solutions of CS·HCl and aqueous solutions of l-AscA and d-AscA are given for comparison. The CD spectra of both diastereomerically-enriched chitosan polysalts are characterized by a positive Cotton effect (curves *4*, *5*). However, there are differences in both the maximum wavelength (λ_0_) of the positive CD band and its intensity, e.g., for CS·HCl·l-AscA λ_0_ = 250 nm, [θ]_250nm_ = 19.4 × 10^5^ deg·mL·dm^−1^·g^−1^, for CS·HCl·*D*-AscA λ_0_ = 240 nm, [θ]_240nm_ = 34.4 × 10^5^ deg·mL·dm^−1^·g^−1^. In the shortwave region, where λ < 225–230 nm and when λ > 275–280 nm, negative values of the specific ellipticity were observed. It is pertinent to note that the Cotton effect with λ_0_ = 253 nm was reported in [5], but in the negative [θ] region, since solutions of N-substituted chitosan ascorbate (the specific isomer of AscA is not indicated in this work) in dimethylsulfoxide were investigated.

The CD spectra of aqueous solutions of l-AscA and d-AscA, like those of CS·HCl·l-(d-)AscA, have a positive ellipticity band at λ_0_ = 250 and 240 nm, but show a higher intensity of the Cotton effect: [θ]_250nm_ = 21.8 × 10^5^ deg·mL·dm^−1^·g^−1^ and [θ]_240nm_ = 39.2 × 10^5^ deg·mL·dm^−1^·g^−1^, respectively (Figure 1a, curves *1*, *2*). Wittine et al. [31] also recorded a positive Cotton effect at λ_0_ = 247.4 nm for a CH_3_CN solution of l-AscA. The shift of the ellipticity maximum for d-AscA towards shorter wavelengths, in comparison with l-AscA, agrees with the absorption spectra of aqueous solutions of both AscA isomers in the near-UV region, e.g., in the UV spectra of l-AscA and d-AscA there is an intense absorption maximum at 265 nm and 258 nm, respectively. It is well known that the UV absorption spectra of AscA are the result of the excitation of the С=С bond and correspond to the π→π* transition. The hypsochromic shift of the absorption maximum is usually caused by an increased acidity of solution due to a change in the equilibrium between the neutral and anionic forms of AscA [32,33], which agrees with the pH of the solutions under study (see Section 2.2.1 Solution Preparation).

The CD spectrum of the aqueous CS·HCl solution shows a negative dichroic absorption with a very weak band at 210 nm, which is responsible for the optical activity of the acetamide chromophore, and a weak, blurred band in the range of 250–260 nm (Figure 1a, curve *3*). The CD spectra of almost completely-deacetylated chitosan were studied [7], and the low intensity of the Cotton effect at 210 nm confirmed a high degree of deacetylation of the CS·HCl sample used.

The appearance of an intense positive dichroic band with a maximum at λ_0_ = 250 nm and 240 nm in the CD spectra of CS·HCl·l-AscA and CS·HCl·d-AscA solutions, as compared to CS·HCl, is probably due to the formation of an aminoascorbate chromophore due to the interaction of protonated auxochromic groups (–NH_3_^+^) of the aminopolysaccharide chain with ascorbate anions. In this case, the decrease in the intensity of the positive CD band of the CS·HCl·l-(d-)AscA solution in comparison with the l-(d-)AscA solution, as well as our IR and NMR spectroscopic analysis of the interaction of CS·HCl with AscA in an aqueous medium [34], indicate the formation of a spatially-close complex between CS·HCl and a resonance-stabilized ascorbate anion. Domard [7] can also be cited as additional evidence of the amino group participation in this interaction. The author shows that an intense Cotton effect with a maximum near λ_0_ = 260–270 nm appears in the initially monotonic CD spectrum upon the formation of the Cu^II^ complex with the amino group at C2 and the hydroxyl group at C3 in the chitosan chain.

Our investigation of the optical activity of individual AscA isomers in the visible spectrum has shown that the aqueous solutions of l-AscA and d-AscA are characterized by opposite values of specific optical rotation [α] and different types of their ORD curves (Figure 1b, curves *1* and *2*). The l-AscA solution shows right-hand rotation (in contrast to the left-hand rotation when λ > 280 nm in the CD spectrum; see Figure 1a, curve *1*) and an anomalous dispersion, since the [α] = *f*(λ) dependence has an inflection point. The values of +[α] in the explored wavelength range do not vary monotonically: they increase with increasing wavelength in the range λ = 280–365 nm, and decrease when λ = 405–710 nm. The d-AscA solution shows left-hand rotation and normal dispersion: the values of −[α] decrease monotonically in magnitude with increasing λ. Note that within λ = 405–710 nm, the solutions of l-AscA and d-AscA have [α] values close in magnitude, and when λ = 280–365 nm the absolute value of [α] of the d-AscA solution is substantially higher than that of the l-AscA solution.

The aqueous solutions of CS·HCl·l-AscA and CS·HCl·d-AscA, like those of l-AscA and d-AscA, are characterized by the opposite direction of the rotation of the polarization plane (rightward and leftward, respectively), and the different character of their dispersion curves (Figure 1b, curves *4*, *5*). However, the optical activity of the CS·HCl salts of l- and d-AscA somewhat differs from the optical properties of the individual l- and d-isoforms of AscA, e.g., the dispersion curve of the CS·HCl·l-AscA solution, as well as that of the l-AscA solution, although referring to the anomalous type, however, shows not only an inflection point on the [α] = *f*(λ) dependence, but also the almost zero value of [α] at λ ~360 nm. The ORD curve of the CS·HCl·d-AscA solution, as well as that of the d-AscA solution, is smooth, but the observed values of −[α] are somewhat larger in magnitude. As in the case of aqueous solutions of the AscA isomers, the CS·HCl·l-AscA and CS·HCl·d-AscA solutions show values of the specific optical rotation close in magnitude in a certain wavelength range (λ ~ 495–710 nm). The ORD of the aqueous CS·HCl solutions (curve *3*) occupies an intermediate position between those curves of the d-AscA and CS·HCl·d-AscA solutions.

The aqueous CS solutions in l-AscA and d-AscA show smooth normal-type ORD curves, right-hand rotation, and differ only in the values of +[α] (Figure 1c, curves *6*, *7*).

According to the available literature data, smooth ORD curves located in the region of negative values of specific optical rotation are observed for chitosan solutions in organic (СН_3_СООН) and inorganic acids (HCl), and acetate buffer (0.33 М СН_3_СООН + 0.2 М СН_3_СOONa) [8,35,36]. Positive [α] values are characteristic of solutions of chitooligomers with the number of elementary units ≤4 and for the monomers of·d-glucosamine hydrochloride and *N*-acetyl-d-glucosamine [35]. The dispersion curves with a positive sign of [α] (both smooth and nonmonotonic) suggest that the interaction of chitosan with l-AscA in aqueous solution differs spatially from both its interaction with d-AscA and with other organic and inorganic acids.

**Figure 2:**
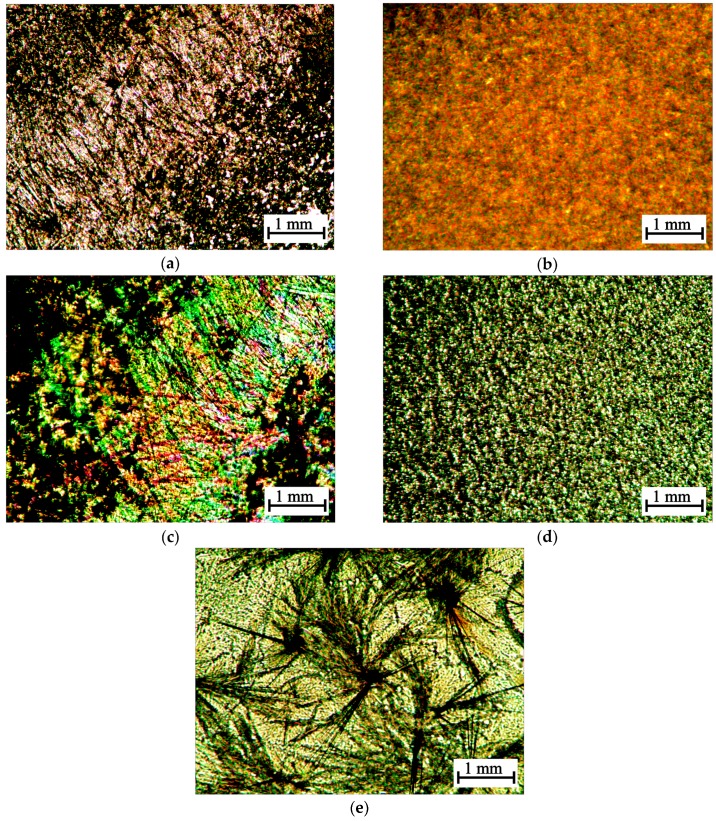
Morphology of the precipitate isolated from the aqueous solution of CS·HCl·l-AscA (**a**,**c**) and CS·HCl·d-AscA (**b**,**d**) of concentrations of 0.5 (**a**,**b**) and 1.0 g·(100 mL)^−1^ (**c**,**d**) with a water-alcohol solution of l-menthol. l-menthol crystals were obtained from its water-alcohol solution of a concentration of 5.0 g·(100 mL)^−1^ by solvent evaporation at 20 ± 2 °C in air (**e**). Polarization microscopy, magnification 10×.

The different nature of the chitosan interaction with l-AscA and d-AscA is confirmed by the results of our study of the morphology of the precipitates isolated from the CS·HCl·l-AscA and CS·HCl·d-AscA solutions with an alcoholic l-menthol solution in polarized light. When CS·HCl·l-AscA is precipitated, predominantly anisodiametric supramolecular structures with pronounced optical anisotropy are formed (Figure 2a,c), when CS·HCl·d-AscA is precipitated, densely-packed confocal domains of a spherical shape are formed (Figure 2b,d). Drying (under the same conditions) of an individual water-alcoholic solution of l-menthol at 20 ± 2 °C leads to the formation of aggregates of needle-shaped particles (Figure 2e).

### 3.2. Formation and Properties of Glycerohydrogels Based on Diastereomerically-Enriched Chitosan Salts

Silicon tetraglycerolate Si(OGly)_4_ solutions in a three-molar excess of GlyOH were used as a precursor to prepare hybrid silicon-chitosan-containing glycerohydrogels. When studying the gelation process, the CS·HCl and CS solutions in l-AscA and d-AscA were mixed with Si(OGly)_4_·3GlyOH in several weight ratios. In all cases, the reaction mixture remained homogeneous during gelation.

**Figure 3:**
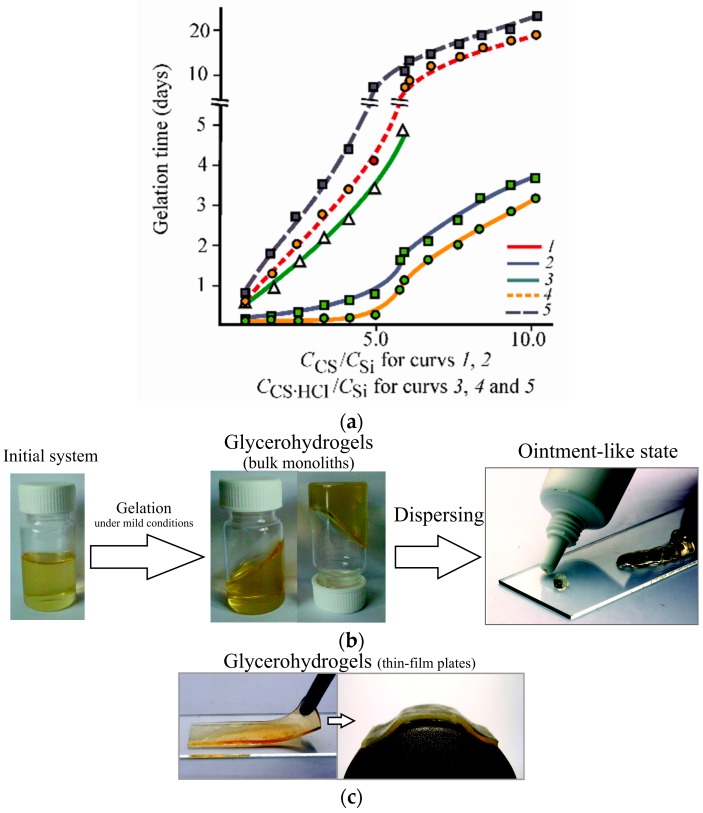
(**a**) Dependence of the gelation time on the *С*_CS_/*С*_Si_ (*С*_CS·HCl_/*С*_Si_) ratio for the systems based on Si(OGly)_4_·3GlyOH and CS·l-AscA (*1*), CS·d-AscA (*2*), CS·HCl (*3*), CS·HCl·l-AscA (*4*), and CS·HCl·d-AscA (*5*) at 20 ± 2 °C. The pH of the initial chitosan solutions with concentrations of 3–4 g·(100 mL)^−1^: рН_CS+l*-*AscA_ = рН_CS+d-AscA_ = 4.2, рН_CS·HCl_ = 3.5, рН_CS·HCl+l*-*AscA_ = рН_CS·HCl+d-AscA_ = 3.1. (**b**,**c**) Photos of our silicon-chitosan-containing glycerohydrogels in the form of bulk monoliths easily passed into an ointment-like state when dispersed (**b**), and in the form of thin-film plates with high congruence to a surface with a complex relief (**c**).

Figure 3a shows the dependence of the gelation time on the weight ratio *С*_CS_/*С*_Si_ (*С*_CS·HCl_/*С*_Si_) for the systems based on CS·l-(d-)AscA (curves *1*, *2*), CS·HCl (curve *3*) and CS·HCl·l-(d-)AscA (curves *4*, *5*) obtained at 20 ± 2 °C. Figure 3b presents an example of the glycerohydrogel thus obtained (it should be noted that all samples of our silicon-chitosan-containing glycerohydrogel easily passed into an ointment-like state when dispersed). It can be seen that for both chitosan samples, and for all the compositions tested, a general tendency of gelation slowing is observed with an increase in the chitosan/precursor ratio, especially pronounced when *С*_CS_/*С*_Si_(*С*_CS·HCl_/*С*_Si_) ≥ 5.0. The longest gelation time was observed with the smallest Si(OGly)_4_ content. It should be noted that the “equimolar” polymer/precursor ratio in the gelling systems under study was realized at *С*_CS_*/С*_Si_(*С*_CS·HCl_*/С*_Si_) ~ 5.5. Since chitosan macromolecules serve as a template for silanol nucleation [15,19,25], an increased polymer content in the system leads to an increased number of “nucleation centers” and a decreased “density” of the distribution of reactive silanol groups which, thus, retards gelation when *С*_CS_*/С*_Si_(*С*_CS·HCl_*/С*_Si_) ≥ 5.0.

The accelerating effect of the polysalt type (mono or binary), the molecular weight of chitosan, and the pH of the gelling medium on the gel point achievement time is also evident. For example, when *С*_CS·HCl_/*С*_Si_(*С*_CS_/*С*_Si_) = 10.0, the CS-l-AscA-based glycerohydrogel (рН_CS+l-AscA_ = 4.2) was formed over 3.1 days, that based on CS·d-AscA (рН_CS+d-AscA_ = 4.2) was formed over 3.9 days (Figure 3a, curves *1*, *2*), and those on the basis of CS·HCl·l-AscA and CS·HCl·d-AscA (рН_CS·HCl+l-AscA_ = рН_CS·HCl+d-AscA_ = 3.1) were formed over 19 and 22 days, respectively (curves *4*, *5*). The glycerohydrogel based on the aqueous CS·HCl solution (рН_CS·HCl_ = 3.5) was formed more slowly than that based on CS·l-(d-)AscA and exceeds CS·HCl·l-(d-)AscA with respect to the gelation rate (curve *3*).

In addition, our experiments on the estimation of the formation kinetics of silicon-chitosan-containing glycerohydrogels using aqueous solutions of diastereomerically-enriched chitosan salts clearly show a significant impact of the chitosan polysalt chirality on the gelation time. In general, for the compositions characterized by the same content of CS·l-(d-)AscA and CS·HCl·l-(d-)AscA, at any *С*_CS_/*С*_Si_(*С*_CS·HCl_/*С*_Si_) ratio, gelation in the system based on chitosan d-ascorbate proceeds for a longer period of time than on the basis of chitosan l-ascorbate (Figure 3a, curves *1* and *2*, *4*, and *5*).

The retardation effect of chitosan d-ascorbate on the gelation kinetics as compared with chitosan l-ascorbate could partly be explained by the different values of the dissociation constant (рК1) of the AscA isomers (рК_l-AscA_ = 4.21 and рК_d-AscA_ = 4.08), which determine the acidity of the medium. However, the pH of the starting solutions of chitosan l-(d-)ascorbate used to prepare glycerohydrogels has the same values: рН_CS+l-AscA_ = рН_CS+d-AscA_ = 4.2, рН_CS·HCl+l-AscA_ = рН_CS·HCl+d-AscA_ = 3.1. Excluding the influence of the pH of the gelling system CS·l-(d-)AscA and CS·HCl·l-(d-)AscA on the gelation kinetics, it was suggested that in this case the spatial organization of diastereomerically-enriched chitosan polysalts with l-, d-ascorbate anions could have a determining effect on the time of formation of the spatial network of the glycerohydrogel. If this is so, then the geometry of the chiral ligand of the polysalt should influence other properties of our glycerohydrogels, for example, the properties of the shape-stable materials obtained therefrom. To verify this hypothesis, physicomechanical characteristics of silicon-chitosan-containing glycerohydrogel thin-film plates were tested. Figure 3c provides an example of such a plate. As can be seen, the glycerohydrogel plate is quite elastic and exhibits high congruence to a surface with a complex relief.

**Figure 4:**
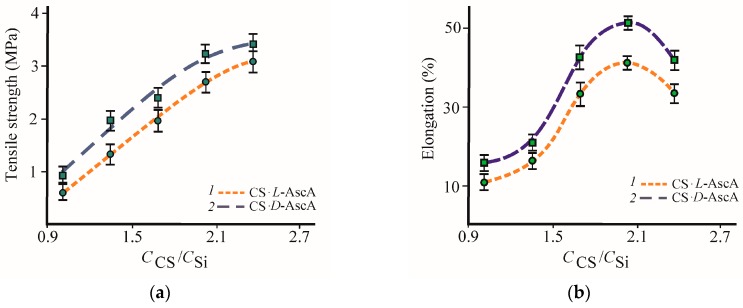

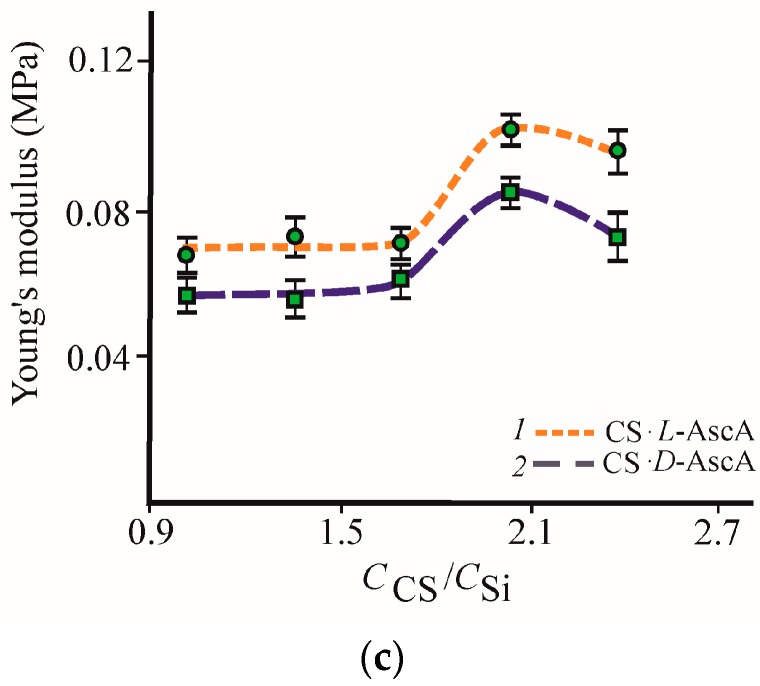
Dependence of the tensile stress (**a**), elongation at break (**b**) and Young’s modulus (**c**) on the *С*_CS_/*С*_Si_ ratio for thin-film plates based on Si(OGly)_4_·3GlyOH, PVA, and CS·l-AscA (*1*), CS·d-AscA (*2*).

Stress-strain curves characteristic of viscoplastic polymeric materials are observed for all samples of our glycerohydrogel plates. With an increase in the *С*_CS_/*С*_Si_ ratio, the tensile strength increases, showing a tendency to reach the maximum values (Figure 4a). The concentration dependencies of elongation at break (Figure 4b) and Young’s modulus (Figure 4c) have an extreme character. Before *С*_CS_/*С*_Si_ ≈ 2.0, the elasticity of our glycerohydrogel plates increases, and then decreases. The increased strength, elongation, and Young’s modulus at break when *С*_CS_/*С*_Si_ ≤ 2 can be due to an increased density of the 3D spatial network of the glycerohydrogel with an increased content of chitosan as the structure-forming component. However, the increased tensile strength and the simultaneously reduced tensile elongation when *С*_CS_/*С*_Si_ > 2.0 were somewhat unexpected, since the increased strength of a thin-film material generally leads to a decrease in its elasticity, and vice versa*.*

All other conditions being equal, the CS·d-AscA-based glycerohydrogel plates are characterized by higher mechanical strength and elasticity under uniaxial stretching and a lower Young’s modulus (Figure 4, curve *1*) as compared with those based on CS·l-AscA (curve *2*). The rupture stress and relative elongation of the plates produced from CS·d-AscA are 20–55% and 25–45% higher than those values for the plates obtained using CS·l-AscA. The lower values of Young’s modulus for the CS·l-AscA plates, in comparison with CS·d-AscA, are quite natural and agree with the nature of supramolecular ordering of diastereomerically-enriched chitosan structures (see Figure 2). For example, the effect of greater stability under uniaxial stretching of the spatial network of globular biopolymer structures due to the entropic nature of their elasticity is known [37,38]. Deformation of such structures is associated with the development of significantly less “elastic” forces as compared with polymeric structures with the fibrillar nature of supermolecular ordering.

Thus, the chirality of the organic ligand (l-, d-ascorbate anion) of diastereomerically-enriched chitosan salts affects not only the gelling kinetics, but also the strength of the spatial network of our silicon-chitosan-containing glycerohydrogels and, accordingly, the concentration dependencies of the elastoplastic parameters of thin-film glycerohydrogel substrates.

### 3.3. Antibacterial Activity of *l*- and *d*-Diastereomerically-Enriched Chitosan Salts

Our microbiological study revealed differences in the antimicrobial activity of the solutions of l- and d-diastereomerically-enriched chitosan salts. As an example, Table 1 shows the antibacterial activity (*Аi*) of the aqueous solutions of CS·HCl·l-AscA and CS·HCl·d-AscA against *S. aureus* 209P and *E. coli* 113-13, expressed in units of the reference concentration of the standard. The values of *Аi* of the aqueous solutions of l-AscA and d-AscA are given for comparison.

A comparison of the values of *Аi* of the individual AscA diastereomers shows that the biocidal activity of the l*-*AscA solution compared to the d-AscA solution is significantly higher for both *E.coli* strain 113-13 (by 1.9 times, *p* ≤ 0.05) and *S. aureus* 209P (by 2.1 times, *p* ≤ 0.05). A different picture was observed when the bacterial cultures were exposed to the solutions of CS·HCl·l-AscA and CS·HCl·d-AscA. It turned out that the CS·HCl·l-AscA solution is 3.2 times and 7.5 times less active against the solution of CS·HCl·d-AscA with respect to *S. aureus* 209P (*p* ≤ 0.05) and *E. coli* 113-13 (*p* ≤ 0.05), respectively.

In comparing the antibacterial activity of the individual diastereomers of AscA and their salts with CS·HCl it was established that the value of *Ai* of d-AscA was statistically significantly lower than that of CS·HCl·d-AscA: 1.7 times and 2.2 times against *S. aureus* 209P and *E. coli* 113-13, respectively. At the same time, the values of *Аi* of l-AscA were significantly higher than those of CS·HCl·l-AscA: 6.3 times and 4.0 times (*p* ≤ 0.05) for *E. coli* 113-13 and *S. aureus* 209P, respectively.

The values of antibacterial activity against the gram-negative strain *E. coli* 113-13 for the solutions of l-AscA and d-AscA were 1.3-fold (*p* ≥ 0.05) and 1.5-fold (*p* ≤ 0.05) higher than those against the Gram-positive *S. aureus* 209P, respectively. No statistically significant differences in sensitivity to the biocidal effect of the CS·HCl·l-AscA solutions were found in the cultures tested. The ratio of *Аi* calculated for *S. aureus* 209P and *E. coli* 113-13 was 1.3 (*p* ≥ 0.05) for this polysalt. At the same time, *E. coli* strain 113-13 showed a greater sensitivity to the influence of the CS·HCl·d-AscA solutions than *S. aureus* 209P—by 1.9 times (*p* ≤ 0.05).

Thus, it has been established that pure l-AscA significantly exceeds pure d-AscA in its ability to inhibit the growth of both Gram-positive and Gram-negative bacteria. However, solutions of its salt with chitosan CS·HCl·l-AscA showed significantly lower antibacterial activity than CS·HCl·d-AscA solutions.

### 3.4. Evaluation of the Viability of NHDF Cells in the Presence of *l*- and *d*-Diastereomerically-Enriched Chitosan Salts

**Figure 5:**
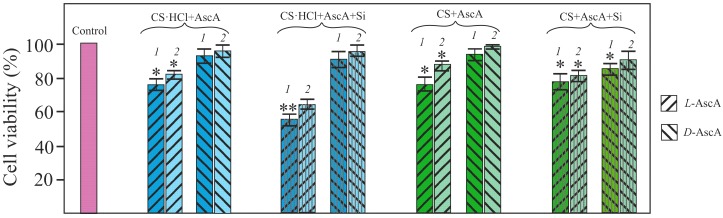
Viability diagram of NHDF cells in the presence of CS·HCl·l-(d-)AscA and CS·l-(d-)AscA without and with Si(OGly)_4_·3GlyOH added, the ratio *С*_CS·HCl_/*С*_Si_(*С*_CS_/*С*_Si_) = 4.8, solutions of a concentration 10^−2^ (*1*) and 7 × 10^−3^ g·(100 mL)^−1^ (*2*) were used. Asterisks indicate statistical significance compared with the control: *p* < 0.05 (*) and *p* < 0.01 (**).

In vitro culturing NHDF cells in the presence of therapeutic doses of solutions and glycerohydrogels of our diastereomerically-enriched chitosan polysalts CS·HCl·l-(d-)AscA and CS·l-(d-)AscA, with and without Si(OGly)_4_·3GlyOH added, showed no cytotoxicity. All systems based on chitosan d-ascorbate statistically significantly promoted the NHDF cell viability in comparison with chitosan l-ascorbate (Figure 5). This result was quite unexpected, since ascorbic acid exhibits biological activity only in the form of its l-isomer.

During in vitro cultivation of NHDF cells in the presence of therapeutic doses of the individual AscA diastereomers (l-AscA and d-AscA) no statistically significant differences in the cell viability were found.

## 4. Conclusions

The revealed differences in the chirality of l- and d-diastereomerically-enriched chitosan ascorbates exert a significant influence on the kinetics of the biomimetic sol–gel synthesis of silicon-chitosan-containing glycerohydrogels. Previously, it was shown that the gelation kinetics of the systems based on chitosan salts and silicon tetraglycerolate was controlled by the acidity of the medium, determined by the nature and acid-base properties of the acid used [19,26,27]. Taking into account the same pH values of the source gelling systems, we associate the retarding or accelerating effect of chitosan ascorbate with the stereoisomerism of the chiral ligand and, correspondingly, with features of the spatial structure of the corresponding polysalt. This assumption is confirmed by the results of our study of the physicomechanical characteristics of silicon-chitosan-containing glycerohydrogel plates and the supramolecular ordering of diastereomerically enriched chitosan structures.

Moreover, a peculiarity of chitosan ascorbates is that they are formed as a result of the interaction of two optically-active chemical substances, namely, a chiral aminopolysaccharide and the l- or d-diastereomer of ascorbic acid. The greatest antibacterial activity of chitosan d-ascorbate and the highest metabolic activity of the NHDF cell culture in the presence of the systems based on chitosan d-ascorbate, as compared to chitosan l-ascorbate, unfortunately have no explanation yet and are currently being investigated. One could only hypothesize that steric features of the organic ligand and the general chiral structure of the polysalt are critical for establishing the full interaction of the protonated amino groups of chitosan with the binding sites on the surface of cell membranes. This should be taken into account when designing biomedical preparations based on chitosan salts.

## Figures and Tables

**Table 1:** Values of the antibacterial activity of aqueous solutions of l-(d-)AscA and CS·HCl·l-(d-)AscA.

Solution	Antibacterial activity (*Ai*), %	
	*Staphylococcus aureus* 209P	*Escherichia coli* 113-13
l-AscA	36.4	45.7
d*-*AscA	17.0	24.6
CS·HCl·l-AscA	9.2	7.3
CS·HCl·d*-*AscA	29.2	54.7
